# Penalization method to convert Bayesian optimization methods into batch multi-objective Bayesian optimization methods

**DOI:** 10.1371/journal.pone.0354346

**Published:** 2026-07-21

**Authors:** Adelle Holder, Henry DeBruin, Jesse M. Sestito

**Affiliations:** College of Engineering, Valparaiso University, Valparaiso, Indiana, United States of America; Northeastern University, CHINA

## Abstract

Bayesian optimization is a surrogate-based global optimization method that is increasingly being used for engineering design. However, most methods are designed to be sequential, and in optimization problems where the functional evaluation can be easily parallelized, batch methods are more effective at reducing real-time computation. While existing batch multi-objective Bayesian optimization (B-MOBO) methods achieve strong performance, they are typically purpose-built from scratch, limiting ability to leverage the extensive library of proven sequential acquisition functions for parallel settings. In this paper, a generalizable transformation methodology is developed that converts compatible single- or multi-objective sequential Bayesian optimization methods into B-MOBO methods. The key innovation is a Euclidean distance-based composite acquisition function with multi-objective penalization averaging, which combines multiple sequential acquisition functions while incorporating objective-wise penalization information from all objectives and preventing redundant sampling in batch selection. To demonstrate the schema, this methodology is applied to two representative sequential acquisition strategies, the expected improvement method (single-objective) and the quality metrics method (multi-objective), to develop two new B-MOBO variants. These new methods are then compared against their sequential counterparts and established B-MOBO methods using both computation time and real-time analysis. Results show that the new B-MOBO methods are as effective as existing methods at solving optimization problems in terms of solution quality and show improvements when real-time is considered.

## Introduction

To solve engineering design optimization problems efficiently, surrogate-based global optimization methods have been developed. Different from population based global optimization, such as particle swarms [1] and genetic algorithms [2], in surrogate-based optimization, a surrogate of the objective is constructed and updated during the searching process. The surrogate is used to identify the most promising region and guide the search. Since the surrogate is much cheaper to evaluate than the original objective, the overall sampling cost is reduced.

One such surrogate-based method is Bayesian optimization (BO). The typical surrogate used in BO is the Gaussian process (GP) model, which also quantifies the uncertainty. In BO, an acquisition function is constructed from the surrogate to identify the candidate sample most likely to improve the objective value, guiding the sequential sampling process. This surrogate-based approach is ideal for optimizing black-box objectives where evaluations are time-consuming and costly [3–8].

Solving single-objective problems with sequential Bayesian optimization (S-BO) has been well explored in design optimization [9–12]. However, in real-world applications, design problems usually involve optimizing multiple competing objectives, such that improving one objective often degrades another. This requires trade-offs between different objectives meaning there is no one optimal value. Instead, there is a set of optimal solutions, or values for the input parameters, that are optimal and form what is called the Pareto front, separating the feasible region from the infeasible one. Multi-objective Bayesian optimization (MOBO) methods have been developed to obtain the Pareto front. In sequential sampling MOBO (S-MOBO), a set of initial samples and their corresponding solutions are passed into the S-MOBO algorithm which recommends a sample that is most likely to exist on the Pareto front. S-MOBO algorithms can be used to discover the Pareto front of the objective space quickly and efficiently [13,14].

One major limitation of S-MOBO methods is their inherently sequential nature. Only one iteration of the design can be built at a time, which increases the real-time required for optimization. In many modern engineering applications, computational resources support parallel evaluation of objective functions through high-performance computing clusters, multi-core processors, or distributed experimental facilities [15], and recent surrogate-assisted methods have been developed to exploit these resources efficiently across a range of optimization problem classes [16,17]. This capability motivated the development of batch multi-objective Bayesian optimization (B-MOBO), which can recommend multiple sets (a batch) of design parameters simultaneously, whose solutions are expected to exist along the Pareto front [3,18,19]. By evaluating multiple candidates in parallel, B-MOBO methods can dramatically reduce real-time computation costs even though the total number of function evaluations may remain similar or slightly increase compared to sequential methods. This makes parallelization essential for time-critical engineering design problems where waiting for sequential evaluations is impractical.

The development of batch methods has progressed through several stages. Early batch approaches for single-objective optimization focused on strategies such as constant liar, Kriging believer [20], and q-Expected Improvement [21]. These methods addressed the fundamental challenge of batch selection on how to choose multiple points when the outcomes of other points in the batch are unknown. Gonzalez *et al.* introduced local penalization as an efficient heuristic for batch generation, using Lipschitz continuity estimates to create local repulsion around selected batch members, thereby preventing redundant sampling [15]. This approach achieved large computational savings compared to more elaborate alternatives. Subsequently, Alvi *et al.* extended local penalization to asynchronous settings, demonstrating that asynchronous methods can outperform synchronous batch approaches in both real-time and sample efficiency [22]. These strategies have since been adapted for multi-objective settings.

While existing B-MOBO methods have been developed, most approaches are either purpose-built B-MOBO methods or individual adaptions of S-MOBO [23–26]. Recent B-MOBO methods employ various strategies including ensemble acquisition functions, hypervolume-based criteria, and diversity-guided selection, as well as advanced surrogate modeling techniques for high-dimensional problems [27,28]. For instance, Daulton *et al.* developed noisy Expected Hypervolume Improvement method which handles noisy objectives and scales batch selection from exponential to polynomial complexity through cached box decomposition [29]. Lin *et al.* proposed parallel multi-fidelity MOBO approaches incorporating strategies for variable-fidelity optimization with correlated objectives [30]. Luković *et al.* introduced diversity-guided B-MOBO [3]. Recently, Ngo *et al.* presented MOBO-OSD, which generates diverse Pareto solutions via orthogonal search directions combined with Pareto front estimation for batch optimization [31].

A wide array of S-BO and S-MOBO methods already exist and are highly effective at discovering the Pareto front [7,32–34]. S-BO methods are effective for varying optimization problems, where selecting the best acquisition function for the problem at hand can reduce the time to discover the true Pareto front. However, while existing B-MOBO methods employ strategies such as local penalization and ensemble acquisition functions, a fundamental gap remains. These methods are typically designed from scratch for batch multi-objective optimization rather than providing a systematic framework to convert existing sequential methods. If compatible S-BO and S-MOBO methods can be turned into B-MOBO methods, these extensive libraries of proven sequential acquisition functions can be leveraged and parallelized for engineering design problems, combining their effectiveness with the advantage of batch selection.

This work addresses this gap by developing a Euclidean distance-based composite acquisition function with multi-objective penalization averaging that transforms compatible single-objective and multi-objective sequential acquisition functions into batch multi-objective methods. Unlike existing local penalization methods that primarily focus on preventing redundant sampling through Lipschitz-based exclusion zones, our penalization averaging approach incorporates objective-wise penalization information from all objectives while maintaining computational efficiency. The resulting schema enables systematic conversion of compatible S-BO and S-MOBO methods into B-MOBO methods without problem-specific modifications. To demonstrate the generality and effectiveness of this schema, we apply it to two well-established methods, the expected improvement method [34] and quality metrics method [35], converting them both to B-MOBO methods. These new methods are then compared to their S-MOBO counterparts as well as against an established B-MOBO method to evaluate the performance gains from parallelization and the competitiveness of the proposed transformation approach.

## Methods

### Proposed batch multi-objective Bayesian optimization transformation algorithm

**Fig 1:**
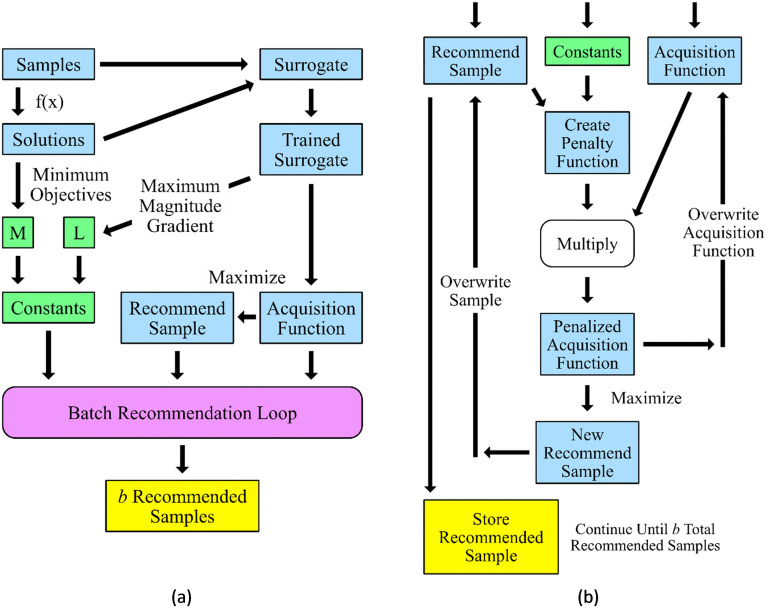
Transformation algorithm for converting S-MOBO methods into B-MOBO methods. The acquisition function is assumed to be the combined acquisition function for S-MOBO. (a) illustrates the overall algorithm while (b) is the Batch Recommendation Loop.

The goal of the proposed B-MOBO transformation algorithm is to convert compatible S-BO and S-MOBO methods into B-MOBO methods. This process first involves the conversion of a S-BO method into a S-MOBO method, followed by the conversion of a S-MOBO method into a B-MOBO method. The transformation algorithm is outlined in Fig 1. A compatible method is defined as one whose acquisition function is non-negative, is computable pointwise from the trained surrogate, and is formulated such that larger acquisition values indicate more promising samples. Acquisition functions that do not satisfy these conditions require modification, such as shifting, before the transformations can be applied.

To understand how the MOBO formation works, it is important to understand the BO formation. The goal of BO is to select a sample ($\text{x}=(x_{1},x_{2},\cdots ,x_{n})$ for *n* input parameters) that minimizes or maximizes, depending on the problem, an objective (*f*(**x**)). To do this, a surrogate that is cheap to evaluate is fit to the existing data to allow for predictions for the objective based on a set of input parameters. This surrogate is then used to create an acquisition function (*a*_1_(**x**)), that is maximized to yield the sample most likely to improve the objective. In this work, all objective functions are minimized while all acquisition functions are maximized, such that larger acquisition values indicate more promising samples.

To convert single-objective optimization methods into multi-objective optimization methods, a new acquisition function must be defined that can be used to select the next sample. First, an acquisition function is developed for each of *m* objectives. Next, the new combined acquisition function (*a*(**x**)) is formulated as the Euclidean distance defined as


$$\begin{array}{c} a(\text{x})=\sqrt{a_{1}(\text{x}{)}^{2}+a_{2}(\text{x}{)}^{2}+\cdots +a_{m}(\text{x}{)}^{2}} \end{array}$$ (1)

where $a_{i}(\text{x})$ is the acquisition function built for the ith objective. The new acquisition function is sufficient to convert compatible S-BO methods into corresponding S-MOBO methods. This formulation can be used for any number of objectives, even one. Hereafter, the following calculations are derived assuming the combined acquisition function.

For the conversion of S-MOBO methods into B-MOBO methods, a penalization method is developed based on a penalization method by González *et al.* which was originally written for single-objective maximization [15]. The González penalty method is adopted and modified to work for the minimization of objective functions as well as multiple objectives. The penalization method for single-objective optimization is defined such that the first new sample **x**_1_ is selected as normal. Each subsequent sample is selected from a penalized acquisition function defined as


$$\begin{array}{c} a_{j}^{\prime }(x)=a(x)\prod_{i=1}^{j-1}\phi_{\text{net}}(x,x_{i}),\;2\leq j \end{array}$$ (2)

where ${\text{x}}_{i}$ is the ith new recommended sample. The net penalty function ($\phi_{net}$) is then the average of each objective’s penalty function such that


$$\begin{array}{c} \phi_{net}=\sum_{k=1}^{m}\frac{\phi_{k}(\text{x},{\text{x}}_{i})}{m} \end{array}$$ (3)

where $\phi_{k}$ is the penalty function for each objective defined as


$$\begin{array}{c} \phi_{k}(x,x_{i})=\frac{1}{2}\mathrm{erfc}(-z_{k}(x,x_{i})) \end{array}$$ (4)

$$\begin{array}{c} z_{k}(x,x_{i})=\frac{L_{k}\|x_{i}-x\|-(\mu (x_{i})-M_{k})}{\sigma (x_{i})\sqrt{2}} \end{array}$$ (5)

where $\mu \left(x_{i}\right)$ and $\sigma (x_{i})$ are the mean and standard deviation for $x_{i}$ predicted by the surrogate trained on the known samples and solutions. $M_{k}$ is the minimum observed value of the *k*th objective and serves as the reference optimum in the penalty. $L_{k}$ is a Lipschitz constant calculated as the maximum gradient magnitude of the *k*th objective’s trained surrogate within the input parameter bounds. Together, $M_{k}$ and $L_{k}$ set the radius of the exclusion zone applied around each previously selected batch member.

To adopt this penalization method for multi-objective optimization, the penalization function can be computed for each objective, such that there are *m* penalization functions. The penalization functions are then combined using a simple average, and then multiplied with the acquisition function. The average is selected over alternatives such as product, minimum, or weighted rules for three reasons. First, the average is invariant to the number of objectives, whereas the product compounds the penalties such that the net penalization strengthens as objectives are added, and the minimum retains only the single most-penalizing objective. As such, a region strongly penalized by one objective but promising for the others is over-excluded under the product and minimum rules, while the average preserves the penalization contribution of every objective and limits the influence of any single one. Second, a weighted average would require objective-specific weights to be specified or tuned for each problem, reintroducing the problem-specific adjustment that the transformation is designed to avoid. Third, for a single objective the average reduces exactly to the penalization of González *et al.* [15], preserving the original single-objective behavior. The simple average is also the most straightforward rule to apply to any acquisition function. *L* becomes $\text{L}=[L_{1},\ldots ,L_{m}]$ and *M* becomes $\text{M}=[M_{1},\ldots ,M_{m}]$. As such, z becomes $\text{z}=[z_{1},\ldots ,z_{m}]$, ϕ becomes $\phi =[\phi_{1},\ldots ,\phi_{m}]$ and the penalized acquisition function becomes


$$\begin{array}{c} a_{j}^{\prime }(x)=a(x)\prod_{i=1}^{j-1}\frac{\sum_{k=1}^{m}\phi_{k}(x,x_{i})}{m},\;2\leq j. \end{array}$$ (6)

In the above, *i* indexes the previously selected batch members that penalize the candidate, *j* indexes the batch candidate currently being selected, and *k* indexes the *m* objectives.

**Fig 2:**
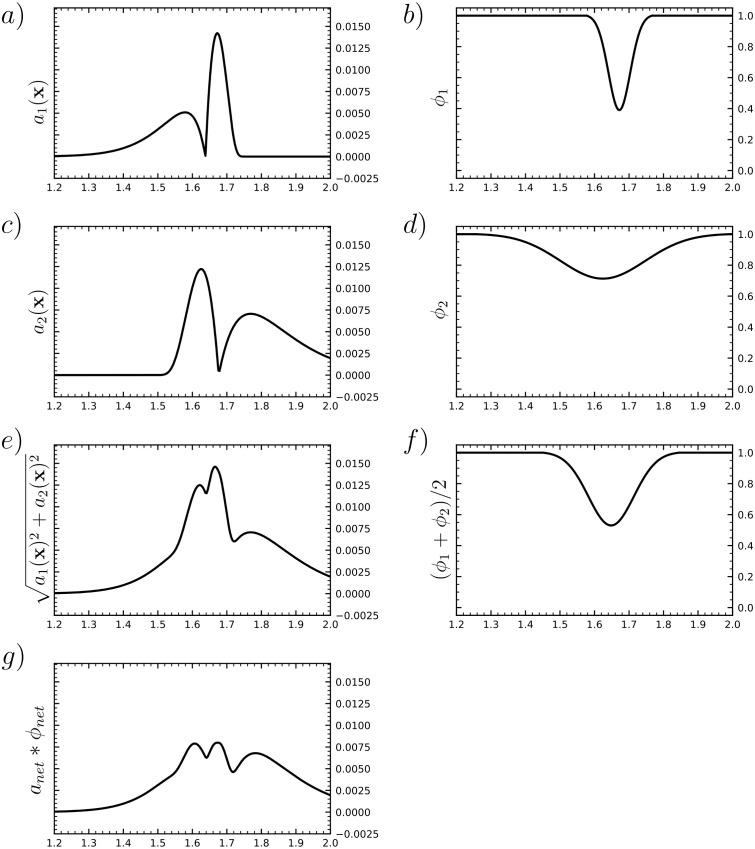
A graphical representation of the transformation algorithm illustrating how a S-BO method is converted to a B-MOBO method. a), c), and e) outline how a S-BO method is converted into a B-MOBO method through the formation of a new acquisition function. b), d), and f) are the corresponding penalty functions for the a), c), and e) acquisition functions. g) is the product of e) and f) to demonstrate the new acquisition function that the next batch sample will be selected from.

A visual representation of S-BO to B-MOBO transformation algorithm is demonstrated in Fig 2. This new penalization acquisition function can now be applied to existing S-BO and S-MOBO methods to create B-MOBO methods.

### Existing Bayesian optimization methods to transform

This methodology is applied to the expected improvement (EI) method [34], which is a S-BO method, and the quality metrics method [35], which is a S-MOBO method, to convert them both into B-MOBO methods.

For the quality metrics method, the relative improvement (*I*) for the quality metrics (QM) was changed from the relative change to the relative difference method defined as


$$\begin{array}{c} I(x)=\left\{ \begin{array}{cc} |\frac{\text{QM}(\{D_{n},x\})-\text{QM}(D_{n})}{(|\text{QM}(\{D_{n},x\})|+|\text{QM}(D_{n})|)/2}|, & \text{QM}(\{D_{n},x\})\neq \text{QM}(D_{n})\\ 0, & \text{QM}(\{D_{n},x\})=\text{QM}(D_{n}) \end{array}\right. \end{array}$$ (7)

where $D_{n}$ are the current n sample points and x is the added sample set. It was changed to the relative difference method to account for quality metrics that can be negative. The quality metrics used for the optimization are the dimensional overall spread (DOS) and integral hyperarea difference (IHD). The DOS is defined by the minimum bounding hyperrectangle that encompasses the current estimated Pareto front. This is also known as a hypervolume with a reference point of the objective-wise minimum of the Pareto points. The IHD is defined as the area under a set of simplexes that estimates the Pareto front [35].

## Results and discussions

To demonstrate the effectiveness of the proposed algorithm, the algorithm is applied to the multi-objective QM optimization method and single-objective EI method to create two new B-MOBO methods. To express the EI method as a multi-objective method in the Euclidean domain, it is henceforth denoted as EIMe. To quantify these new B-MOBO methods, the numerical examples ZDT1, ZDT2, ZDT3, FON [36,37], and DTLZ2 [38] and a welded beam engineering design problem [39] are adopted for optimization. To evaluate the effectiveness of the new methods, several comparisons will be made. First, each new B-MOBO method will be compared to their S-MOBO counterparts, henceforth known as a batch size of 1. Second, the new B-MOBO methods will be compared to the B-MOBO Ensemble method by Lyu *et al.* [14] and the q-Expected Hypervolume Improvement (qEHVI) method by Daulton *et al.* [29]. All Bayesian optimization methods use a Gaussian surrogate with a Matérn 5/2 kernel to balance smoothness and modeling flexibility. Kernel hyperparameters are optimized via maximum likelihood estimation with ten random restarts. These settings follow standard practices in the Bayesian optimization literature [40,41] and are held fixed across all experiments to ensure fair comparisons. The qEHVI method is implemented using the BoTorch library. Its acquisition function is optimized using multi-start L-BFGS-B with 20 restarts and 1024 raw samples, and the reference point is set to the objective-wise minimum of the observed data offset by 10% of each objective’s range. All methods are executed on comparable computational resources using identical initial sample sets and evaluation budgets.

A performance test is developed to systematically quantify each method for each optimization example and is executed for each optimization method, at each batch size (1, 2, and 3), and for each numerical example. To account for statistical randomness, the performance test is the optimization routine executed 30 times, and the optimization routine is defined as follows: An initial set of 30 samples are created using the Latin Hypercube Sampling [42,43] method and the samples’ corresponding solutions are computed from the functional evaluation. Next, the MOBO method is used to select the next sets of samples corresponding to its batch size: a batch size of one results in one set, a batch size of two results in two sets, and a batch size of three results in three sets. The corresponding solution is calculated for each sample set resulting in one, two, or three new samples and solutions. This data is combined with the original data, the optimization method is used again to recommend the next sample or samples, and the process repeats until a total of 330 samples and solutions are created.

To quantify the performance test, distinct performance metrics are calculated for each of the 30 runs. These metrics are the number of points on the Pareto front (nP), the Pareto quality metric dimensional overall spread (DOS), and the Pareto quality metric integral hyperarea difference (IHD). The mean, standard deviation, and range are calculated for each performance metric. In general, a higher nP is desired and DOS and IHD values closest to the ideal values are desired.

To compare batch and sequential methods, the first type of comparison that will be used is a computation time comparison. To keep computation time constant, function evaluations must be consistent requiring the exact same number of samples to be compared. To illustrate this comparison, let us look at a sequential method versus a batch method of size two. For the sequential method, 30 initial samples are generated, then the optimization is executed 300 times to create a total of 330 samples. For the batch method of size two, 30 initial samples are generated, then the optimization is executed 150 times to create a total of 330 samples. In this instance, both algorithms created 330 samples with 330 functional evaluations and took an equivalent amount of computation time. However, the sequential method required the 300 functional evaluations to be executed in series while the batch method allowed the 300 functional evaluations to be executed in parallel for sets of two. This type of analysis is good for examining the computational time benefits between methods, but falls short when looking at the real-time benefits which batch methods thrive in. The computation time analysis also isolates the two components of the transformation. At a batch size of 1, the penalization product in Eq. 2 is empty, so the results reflect the composite acquisition function alone. Comparing a batch size of 1 against batch sizes of 2 and 3 under the computation time analysis, where the total evaluation budget is matched, therefore isolates the effect of the multi-objective averaged penalization.

To examine the real-time benefits between batch and sequential methods, the number of optimizations is held constant to keep the real-time constant. To illustrate this comparison, a sequential method versus a batch method of size two is used. For the sequential method, 30 initial samples are generated, then the optimization is executed 100 times to create a total of 130 samples. For the batch method of size two, 30 initial samples are generated, then the optimization is executed 100 times to create a total of 230 samples. In this instance, assuming parallelization of functional evaluations, both methods will take an equivalent amount of real-time. It is important to note that the real-time analysis is a parallel-resource budget comparison rather than a direct algorithmic fairness comparison. Under this framing, batch methods with a batch size greater than one access more total functional evaluations within the same wall-clock window, which is the intended advantage of batch methods in applications where evaluations parallelize. The purpose of the real-time analysis is therefore to compare methods at matched batch sizes, that is, the new B-MOBO methods against existing B-MOBO methods, and to show how each method exploits available parallel compute. Differences observed across batch sizes within a single method should not be interpreted as intrinsic methodological superiority. To look at the benefits of the new batch methods, both the computation time and real-time comparisons will be utilized.

### Two-dimensional numerical examples

The two-dimensional numerical examples are defined in Table 1. All numerical examples are executed with the dimension of input variables as *n* = 3.

**Table 1:** Numerical example problems used to test the different multi-objective Bayesian optimization methods.

Test Problem	Formulation
ZDT1	$f_{1}(x)=x_{1}$
	$f_{2}(x)=g(x)\times \left(1-\sqrt{\frac{x_{1}}{g(x)}}\right)$
	$g(x)=1+\frac{9}{n-1}\sum_{i=2}^{n}x_{i}$
	$0\leq x_{i}\leq 1,i=1,...,n$
ZDT2	$f_{1}(x)=x_{1}$
	$f_{2}(x)=g(x)\times \left(1-{\left(\frac{x_{1}}{g(x)}\right)}^{2}\right)$
	$g(x)=1+\frac{9}{n-1}\sum_{i=2}^{n}x_{i}$
	$0\leq x_{i}\leq 1,i=1,...,n$
ZDT3	$f_{1}(x)=x_{1}$
	$f_{2}(x)=g(x)\times \left(1-\sqrt{\frac{x_{1}}{g(x)}}-\frac{x_{1}}{g(x)}\sin(10\pi x_{1})\right)$
	$g(x)=1+\frac{9}{n-1}\sum_{i=2}^{n}x_{i}$
	$0\leq x_{i}\leq 1,i=1,...,n$
FON	$f_{1}(x)=1-\exp\left(-\sum_{i=1}^{3}{\left(x_{i}-\frac{1}{\sqrt{3}}\right)}^{2}\right)$
	$f_{2}(x)=1-\exp\left(-\sum_{i=1}^{3}{\left(x_{i}+\frac{1}{\sqrt{3}}\right)}^{2}\right)$
	$-4\leq x_{i}\leq 4,i=1,2,3$

The ZDT1 problem is first examined to demonstrate the different Bayesian optimization methods. The ZDT1 test problem’s Pareto front can also be analytically solved as [44]


$$\begin{array}{c} f_{2}=1-\sqrt{f_{1}}. \end{array}$$ (8)

From the analytical solution of the Pareto front, the ideal quality metrics are determined to be DOS = 1 and IHD = 1/3.

**Fig 3:**
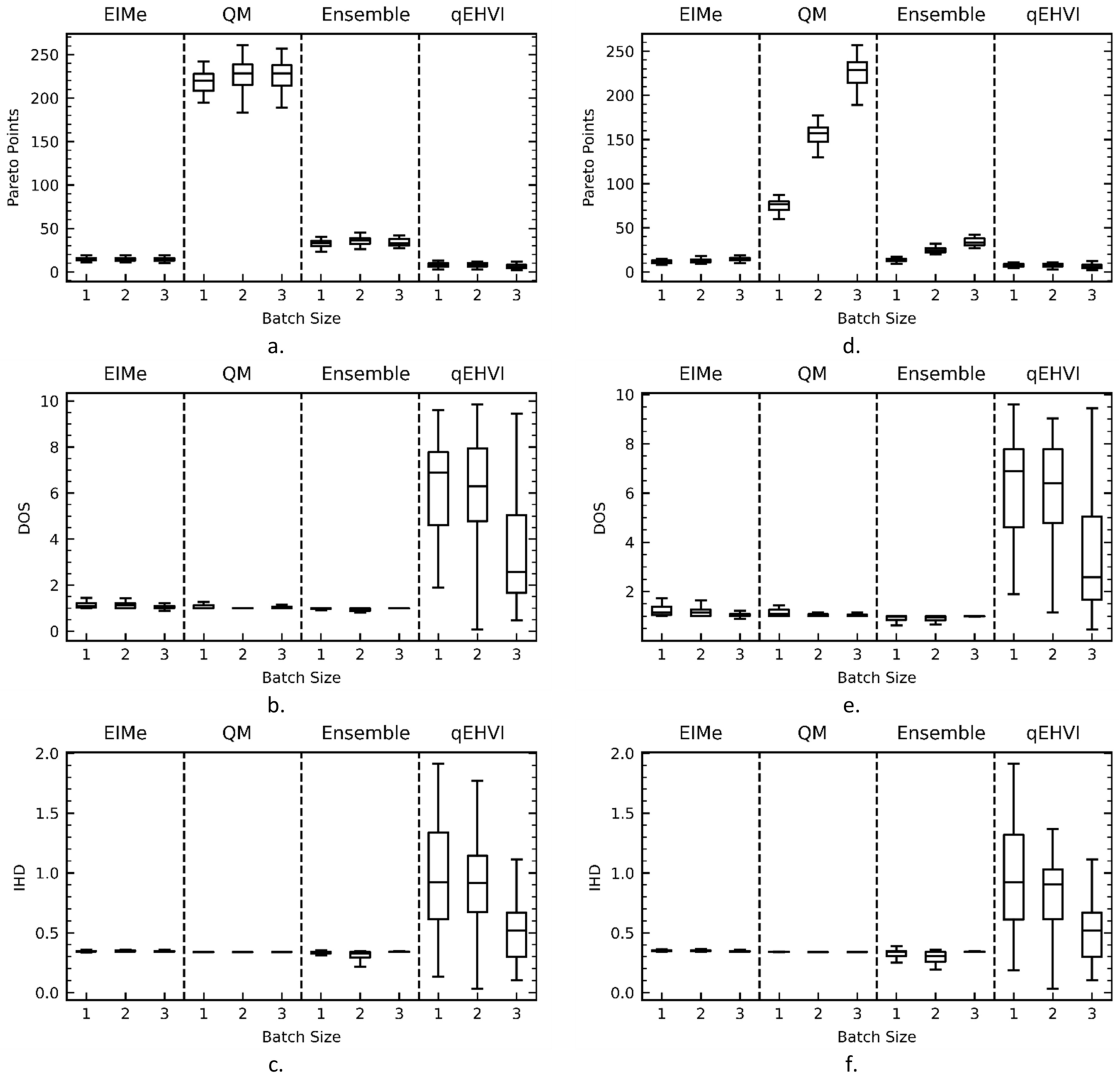
The ZDT1 performance test data. a., b., and c. are generated using a computation time analysis while d., e., and f. are generated using a real-time analysis. A larger number of Pareto points indicates better performance, and dimensional overall spread (DOS) and integral hyperarea difference (IHD) values closer to the ideal values for the problem indicate better performance.

To examine the ZDT1 performance test data, the performance metrics for each of the 30 samples are plotted as box-whisker’s plots with outliers removed. An outlier is determined to be outside a ± 2.698 times standard deviation. Fig 3 a – c has the computation time analysis while Fig 3 d – f has the real-time analysis.

For the computation time analysis of the ZDT1 problem (Fig 3 a – c), we would expect the batch size of 1 to outperform the batch size of 2 and 3. This is because each new sample in the batch size of 1 uses all previous data to inform the next selected sample, which is not the case for the other batch sizes. For ZDT1 case, this is not observed as all batch methods appear to perform similarly with their respective optimization method. This could be due to the simplicity of the ZDT1 example. It is also apparent that each optimization method captures the ZDT1 Pareto front with different success. When examining the number of Pareto points, the QM method is the best, followed by Ensemble, followed by EIMe and qEHVI which show similar results. If looking at DOS, the QM method is the best where the qEHVI overestimates, the EIMe overestimates, and the Ensemble method underestimates. Overall, under the tested ZDT1 setting, the proposed batch variants did not show a clear performance disadvantage relative to their sequential counterparts when computation time is considered.

For the real-time analysis of the ZDT1 problem (Fig 3 d – f), the higher batch size produces a better approximated Pareto front than the lower batch size, as expected when parallel evaluation is available. For the number of Pareto points for the QM method, a batch size of 3 has more Pareto points than a batch size of 1. For all three methods, the DOS and IHD values all converge to the ideal values as batch size is increased. When considering real-time under parallel evaluation, the higher batch size makes more effective use of the available parallel evaluation resource within the same wall-clock window, and an optimization method more suited to solving the ZDT1 problem, such as the QM method, can be selected using the new schema.

The ZDT2 problem also has an exact solution for the Pareto front as


$$\begin{array}{c} f_{2}=1-f_{1}^{2}. \end{array}$$ (9)

This yields an optimal DOS that equals 1 and the optimal IHD of 2/3. The ZDT3 solution [36] is a bit more complex, where $f_{1}\in F$ and


$$\begin{array}{ccc} F & = & \left[0,0.0830]\cup (0.1822,0.2578]\cup (0.4093,0.4539\right]\\ & & \cup (0.6184,0.6525]\cup (0.8233,0.8518] \end{array}$$ (10)

and


$$\begin{array}{c} f_{2}=1-\sqrt{f_{1}}-f_{1}\sin(10\pi f_{1}). \end{array}$$ (11)

This yields an optimal DOS of approximately 1.51 and an optimal IHD of 0.73. The ideal parameters for FON are DOS = 1 and IHD = 2/3 [37].

**Fig 4:**
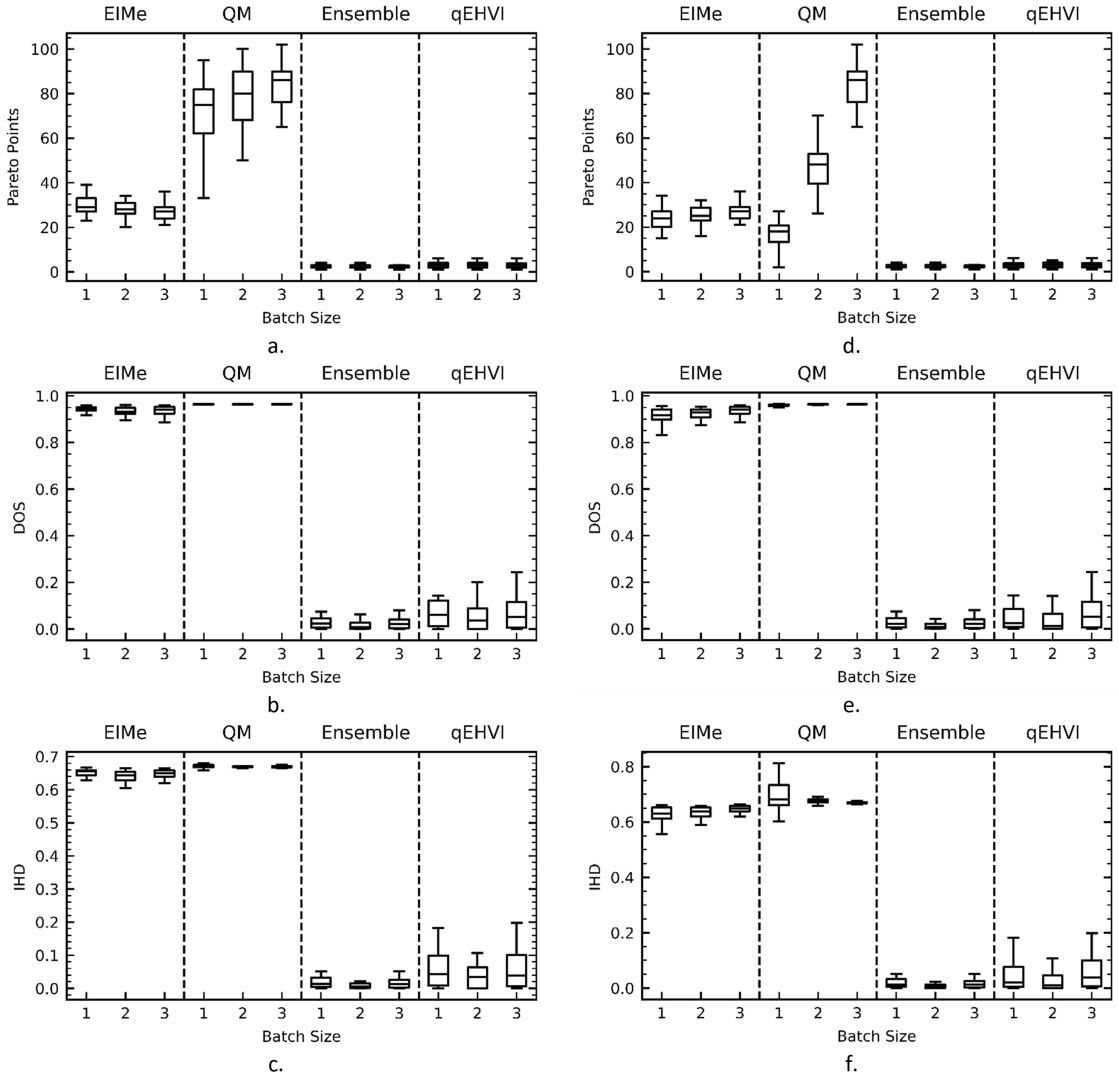
The FON performance test data. a., b., and c. are generated using a computation time analysis while d., e., and f. are generated using a real-time analysis. A larger number of Pareto points indicates better performance, and dimensional overall spread (DOS) and integral hyperarea difference (IHD) values closer to the ideal values for the problem indicate better performance.

For FON, a more complicated two-dimensional Pareto front, similar results as the ZDT1 test problem are observed. When examining the computation time analysis (Fig 4 a – c), it is again observed that the batch methods perform similarly to the sequential method for their respective optimization method. Specifically, for the EIMe, Ensemble, and qEHVI methods, the batch methods are slightly worse. Again, this is expected since the sequential method uses all previous information to guide the sample selection process. However, when going from a computation time analysis to a real-time analysis (Fig 4 d – f), all methods produce better approximated Pareto fronts as larger batches complete more functional evaluations under the parallel-resource budget, with the QM method seeing the largest improvement.

Additionally, when looking at the computation time analysis (Fig 4 a – c), there are large discrepancies between the approximated Pareto fronts calculated using different methods. Even though the EIMe and QM methods have similar DOS and IHD values, the number of Pareto points the QM method has is over double the amount the EIMe method calculates. Additionally, the Ensemble and qEHVI methods perform poorly compared to both the EIMe and QM methods. This indicates that it is important to choose the best BO method for the problem.

The observations on the ZDT2, ZDT3, and FON problems further suggest that when a functional evaluation can be parallelized, the final approximated Pareto front can see improvements over traditional sequential optimization within the same wall-clock window. Additionally, the type of optimization method plays a large role in the established approximated Pareto front, as shown with FON. The ability to select the optimization method most optimal for a specific problem plays a key role in finding strong Pareto fronts. Coupling the correct optimization method with parallelization can result in an improved approximated Pareto front. This is why the ability to turn any compatible sequential optimization method into a B-MOBO method instead of relying on only developed B-MOBO methods can be a powerful tool for optimization problems.

### Higher-dimensional numerical examples

Multi-objective optimization is not just for two-dimensional optimization. These methods must be able to scale to higher dimensions. In this work, these methods are tested with up to five objectives. The QM has difficulties with four or more objectives and is partially excluded from this analysis. The qEHVI method has difficulties with three or more objectives and is excluded from this analysis. To accomplish three to five objective problems, the numerical example DTLZ2 is used. Here, DTLZ2 is formulated to minimize m objectives using n input parameters [38]


$$\begin{array}{cc} f_{1}(x) & =(1+g(x))\cos\left(\frac{x_{1}\pi }{2}\right)\ldots \cos\left(\frac{x_{m-2}\pi }{2}\right)\cos\left(\frac{x_{m-2}\pi }{2}\right)\\ f_{2}(x) & =(1+g(x))\cos\left(\frac{x_{1}\pi }{2}\right)\ldots \cos\left(\frac{x_{m-2}\pi }{2}\right)\sin\left(\frac{x_{m-2}\pi }{2}\right),\\ f_{3}(x) & =(1+g(x))\cos\left(\frac{x_{1}\pi }{2}\right)\ldots \sin\left(\frac{x_{m-2}\pi }{2}\right),\\ & \ldots \\ f_{m}(x) & =(1+g(x))\sin\left(\frac{x_{1}\pi }{2}\right), \end{array}$$ (12)

where $g(x)=\sum_{j=m}^{n}(x_{j}-0.5{)}^{2}$ and $0\leq x_{i}\leq 1,\text{ for }i=1,...,n$. The ideal Pareto solutions exists when $x_{m}$ through $x_{n}$ equal 0.5. The Pareto front is the surface of a unit radius *m*-sphere centered at the origin existing in the first orthant. This results in an optimal DOS of 1 and IHD of $1/2^{m}$ of the hypervolume of the *n*-sphere of radius 1. The worst possible guesses results in an *n*-sphere of radius 1.25+0.25(n−m), and the worst possible DOS and IHD are $(1.25+0.25(n-m){)}^{m}$ and $1/2^{m}$ of the hypervolume of the *n*-sphere of radius 1.25+0.25(n−m). If the Pareto front has a low diversity, the DOS and IHD calculations can be lower than the optimal values.

**Fig 5:**
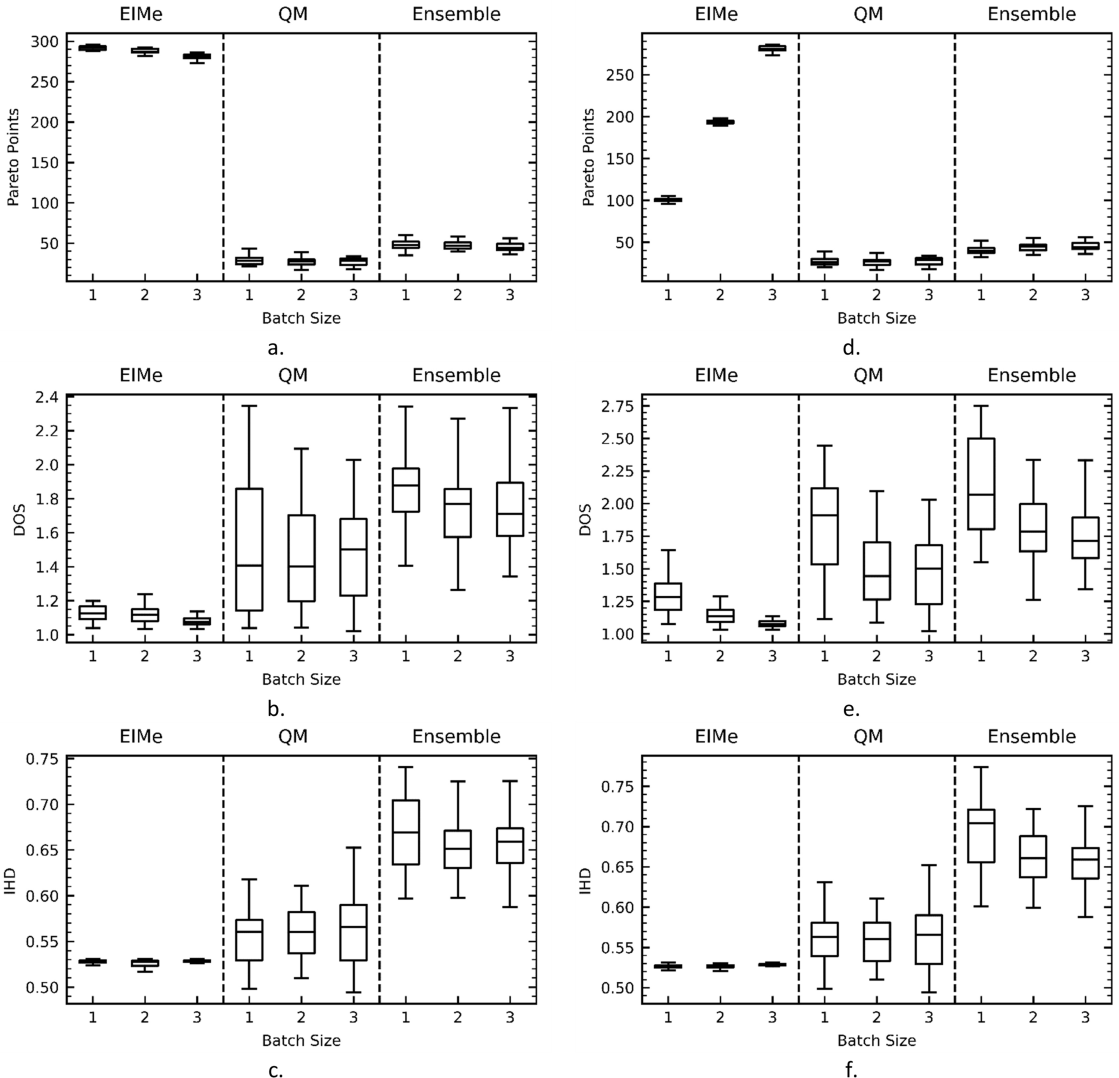
The DTLZ2 performance test data with 3 objectives. a., b., and c. are generated using a computation time analysis while d., e., and f. are generated using a real-time analysis. A larger number of Pareto points indicates better performance, and dimensional overall spread (DOS) and integral hyperarea difference (IHD) values closer to the ideal values for the problem indicate better performance.

**Fig 6:**
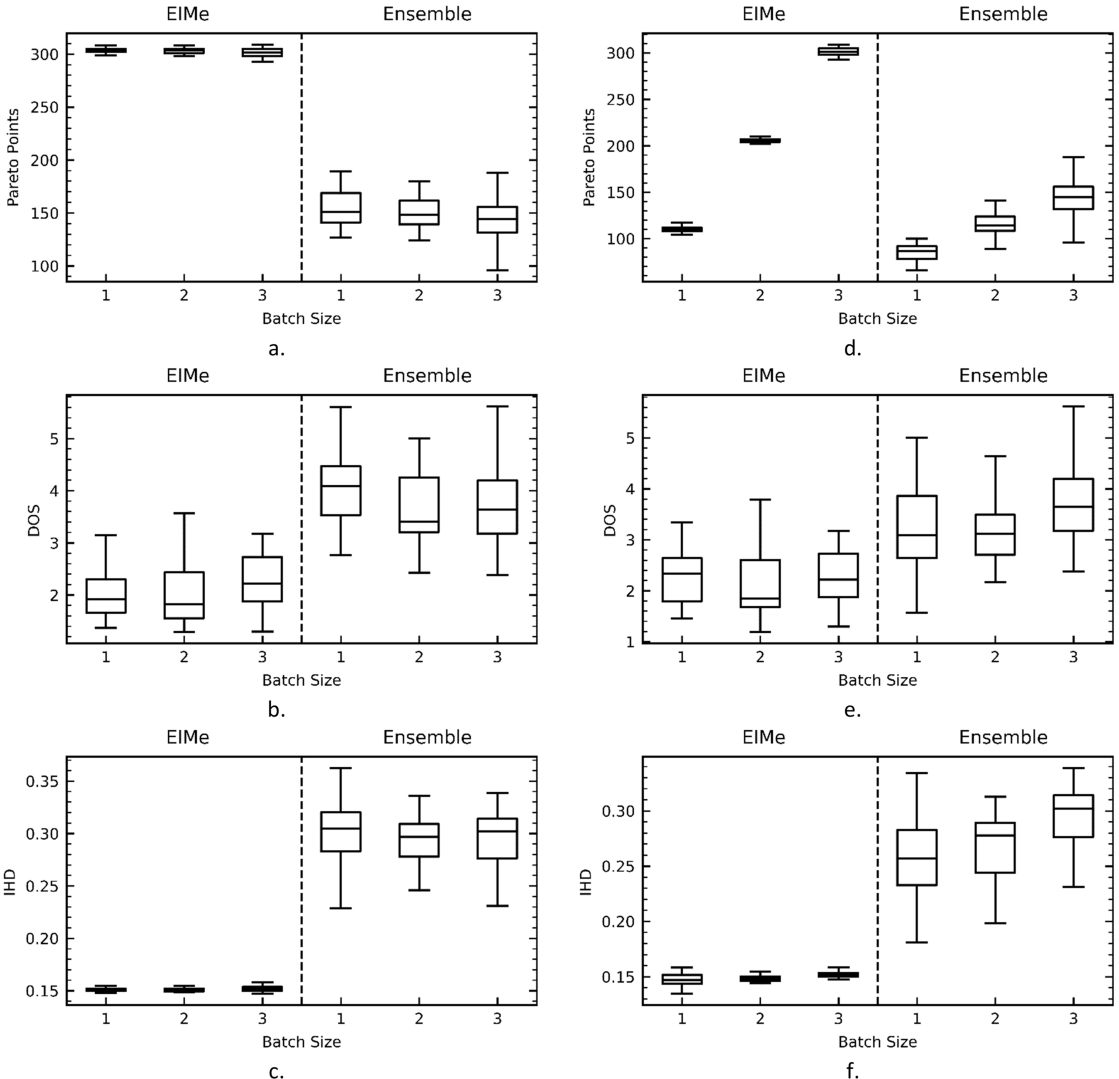
The DTLZ2 performance test data with 5 objectives. a., b., and c. are generated using a computation time analysis while d., e., and f. are generated using a real-time analysis. A larger number of Pareto points indicates better performance, and dimensional overall spread (DOS) and integral hyperarea difference (IHD) values closer to the ideal values for the problem indicate better performance.

The DTLZ2 test problem is executed with *m* = 3 and *n* = 4 following the same execution settings as the two objective numerical examples. In three objective space, the optimal DOS and IHD are 1 and π/6 respectively. The worst possible DOS and IHD are 3.375 and 9π/16 respectively. The EIMe method performs the best. The QM method and Ensemble method both perform well. QM method calculates the quality metrics better than the Ensemble method and the Ensemble method gets more Pareto points than the QM method as seen in Fig 5. Like the two-dimensional problems, different optimization methods are effective for different problems. When looking at the real-time analysis (Fig 5 d – f), the EIMe method, which performed the best, sees improvements from the additional parallel evaluations. The QM and Ensemble methods see slight improvements. When the number of objectives is increased to 5 (Fig 6), similar trends are observed. The EIMe method performs better than the Ensemble method and both methods see improvements under the parallel-resource budget while showing minimal losses in the computation time analysis.

### Engineering example

To compare the methods to an engineering problem, the engineering design of a welded beam that has been employed previously by Ray *et al.* [39] is used. This is a four input variables (*n* = 4) and two objective problem. With a load of 6,000 pounds, length of 14 inches, modulus of elasticity of 30 Msi, the objectives to minimize fabrication cost (*f*_1_) and end deflection (*f*_2_) and constraints shear stress in weld (*g*_1_), bending stress in beam (*g*_2_), geometric constraint of weld thickness less than beam width (*g*_3_), and buckling load constraint (*g*_4_) are


$$\begin{array}{cc} f_{1}(\vec{x}) & =1.10471h^{2}l+0.04811tb(14+l)+P(\vec{x}),\\ f_{2}(\vec{x}) & =\frac{2.1952}{bt^{3}}+P(\vec{x}),\\ g_{1}(\vec{x}) & =\tau -13600,\\ g_{2}(\vec{x}) & =\sigma -30000,\\ g_{3}(\vec{x}) & =h-b,\\ g_{4}(\vec{x}) & =6000-P_{c}, \end{array}$$ (13)

where $\vec{x}=\{h,\;l,\;t,\;b\}$ which correspond to weld thickness, weld length, beam height, and beam width all in inches and τ, σ, and $P_{c}$ are the shear stress in psi, bending stress in psi, and buckling load in pounds respectively. The constraints are implemented as a penalty ($P\left(\vec{x}\right)$) function as adapted from Coello *et al.* [45] as


$$\begin{array}{c} P(\vec{x})=\max(g_{1}(\vec{x}),g_{2}(\vec{x}),g_{3}(\vec{x}),g_{4}(\vec{x}),0). \end{array}$$ (14)

**Fig 7:**
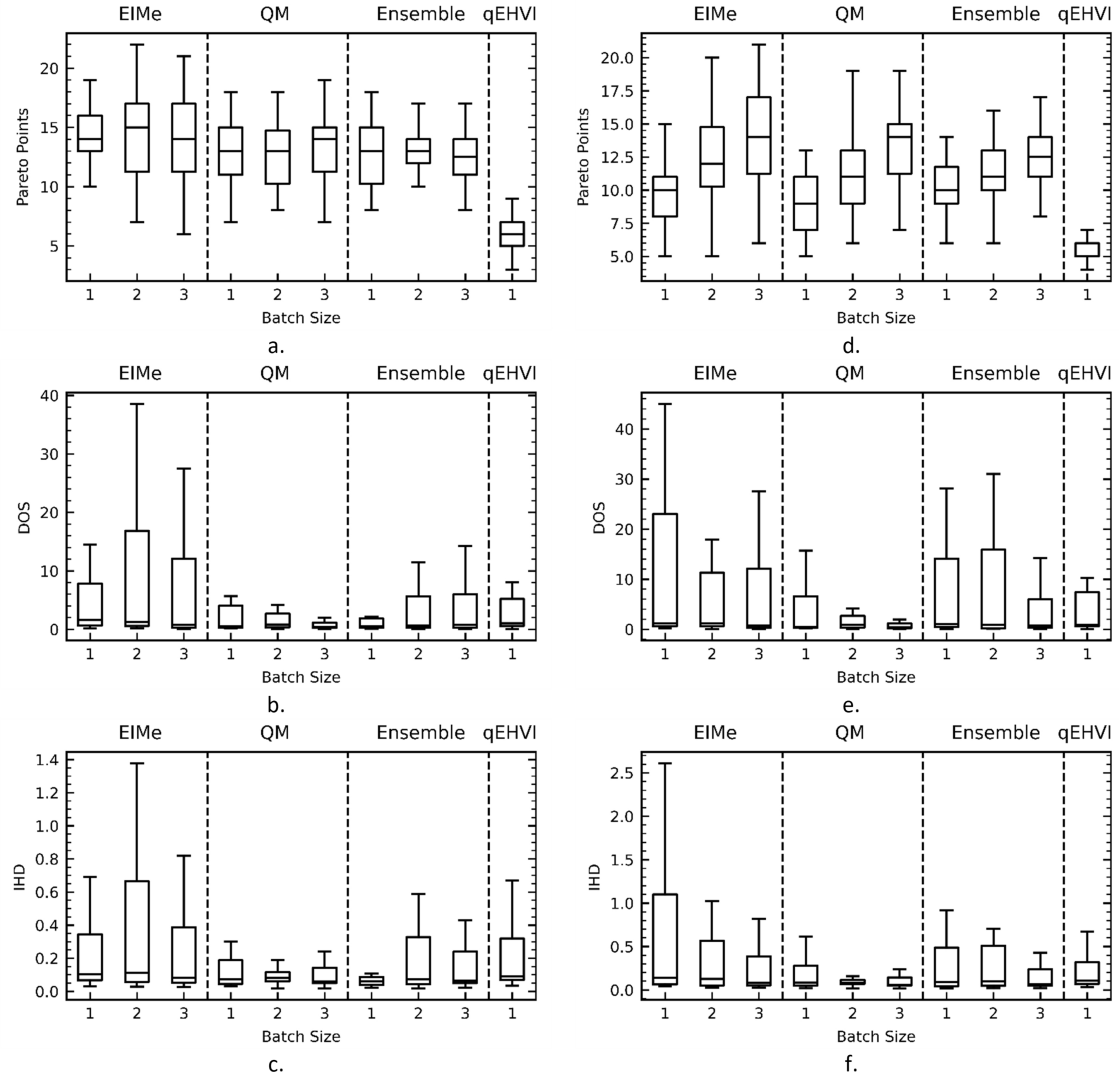
The Welded Beam engineering design problem performance test data with 4 parameters and 2 objectives. a., b., and c. are generated using a computation time analysis while d., e., and f. are generated using a real-time analysis. A larger number of Pareto points indicates better performance, and dimensional overall spread (DOS) and integral hyperarea difference (IHD) values closer to the ideal values for the problem indicate better performance.

The engineering application yields results consistent with the numerical examples as seen in Fig 7. Real-time analysis reveals comparable performance across batch sizes, with smaller batches showing marginal advantages. The computation time analysis, however, demonstrates an advantage for larger batch sizes. Notably, when applying the qEHVI method to the welded beam problem, the acquisition optimization failed for all batch sizes greater than one. Because the penalty is added equally to both objectives, infeasible designs collapse onto a near-degenerate front that ill-conditions the qEHVI hypervolume box decomposition for batch sizes greater than one. These observations underscore the key advantages of the proposed methodology. By enabling the conversion of a compatible S-BO or S-MOBO method into a B-MOBO method, users gain the flexibility to parallelize sampling when functional evaluations support parallelization, while simultaneously selecting the optimization approach best suited to their specific application.

### Batch generation time analysis

To examine the computational efficiency of the transformation algorithm, an experiment is designed to evaluate each method on the ZDT1 benchmark problem with batch sizes ranging from 1 to 3. Additionally, the EIMe method is evaluated with batch sizes from 1 to 10 to assess scalability. For each method and batch size combination, 30 runs were conducted. Each run began with 30 initial samples with an additional 300 samples calculated using the method. Average time per new sample is then calculated. The results in Table 2 reveal distinct computational scaling behaviors across methods. For the EIMe method, the computation time per sample appears to decrease with larger batches, while qEHVI shows an increase as the batch size increases. The other two methods show relatively stable per-sample times. One-way ANOVA confirms that there are significant differences between batch sizes on time per sample for all methods (*p* < 0.001 for all methods). However, upon further examination, linear regression reveals more distinct patterns. Time per sample decreases significantly with batch size for the EIMe method (*p* = 0.004), shows no significant relationship for the QM and Ensemble methods (*p* = 0.494 and 0.178 respectively), and increases significantly for the qEHVI method (*p* = 0.010).

**Table 2:** New sample computation time comparison for the new and established B-MOBO methods.

Method	Batch Size	Average Time per Sample, [s]	Standard Deviation, [s]	Number of Samples
EIMe	1	0.40	0.21	9000
EIMe	2	0.58	0.18	4500
EIMe	3	0.44	0.14	3000
EIMe	4	0.35	0.11	2250
EIMe	5	0.32	0.11	1800
EIMe	6	0.29	0.11	1500
EIMe	7	0.27	0.12	1290
EIMe	8	0.31	0.30	1140
EIMe	9	0.25	0.13	1020
EIMe	10	0.25	0.14	900
QM	1	3.35	3.05	9000
QM	2	3.58	3.03	4500
QM	3	2.70	1.89	3000
Ensemble	1	23.64	4.46	9000
Ensemble	2	11.97	2.27	4500
Ensemble	3	8.05	1.55	3000
qEHVI	1	1.91	0.36	9000
qEHVI	2	2.60	0.67	4500
qEHVI	3	3.33	1.18	3000

The decreasing or stable per-sample time observed for the transformation-based methods (EIMe and QM) is consistent with an observed empirical scaling trend on the order of *O*(*b*) or better, where *b* is the number of samples in the batch. The trends reported here are empirical observations from the timing experiment rather than proven complexity bounds. The per-batch-member computational cost does not appear to grow with batch size for the transformation-based methods, in contrast to the increasing per-sample cost observed empirically for qEHVI. When examining batch sizes up to 10 for the EIMe method, the empirical trend persists, indicating that the transformation approach remains efficient at larger batches in the timing experiment. The decreasing per-sample time observed for EIMe is consistent with amortizing certain calculations across batch members, which makes the schema attractive for applications requiring large batch sizes.

## Conclusion

In this work, a methodology was developed to convert compatible sequential single-objective and multi-objective Bayesian optimization methods into batch multi-objective Bayesian optimization methods. The methodology was demonstrated on two representative sequential acquisition strategies, the expected improvement method and the quality metrics method, and the resultant batch methods were compared to their sequential counterparts and established B-MOBO baselines. When computation time is considered, the batch methods perform slightly worse than their sequential counterparts under the tested settings but remain competitive. When real-time is considered and functional evaluations can be parallelized, the batch methods access more total functional evaluations within the same wall-clock window and produce better approximated Pareto fronts than their sequential counterparts under this parallel-resource budget. This trend was observed for 2, 3, 4, and 5 objectives under the tested settings.

Depending on the functional evaluations and the problem being solved, different Bayesian optimization methods are recommended. As such, it is important to select the best Bayesian optimization method for the problem. Since this new methodology can convert compatible sequential Bayesian optimization methods to batch multi-objective methods, the most appropriate method for a given problem can be converted into a batch method using the new schema. Batch methods should only be used when the functional evaluations can be easily parallelized. When working with functional evaluations which must be sequential, a non-batch method is better.
